# The Contribution of a “Supportive Community” Program for Older Persons in Israel to Their Offspring Who Are Primary Caregivers

**DOI:** 10.1155/2015/914543

**Published:** 2015-08-09

**Authors:** Ahuva Even-Zohar

**Affiliations:** School of Social Work, Faculty of Social Sciences, Ariel University, 40700 Ariel, Israel

## Abstract

The “supportive community” programs in Israel provide a basket of services for older persons living in their own homes. This study examined the differences between caregiver burden and quality of life of 55 offspring who were the primary caregivers of their older parents who were members of a supportive community, compared to 64 offspring whose parents were nonmembers. The findings showed that the role stress factor of caregiving burden was lower, and the psychological health domain of quality of life was higher among offspring whose parents were members of supportive communities. Some of the predictor variables of burden were income status of caregiver, sharing with others in caregiving, and membership of the parent in a supportive community. The primary predictor variable of the quality of life was caregiving burden. The practical conclusion of this study is to further develop and market supportive community programs in various communities.

## 1. Introduction

The current trend in recent years for older adults in Israel as well as in other countries is “aging in place,” which refers to older adults living independently in their current residence or community for as long as possible [1, 2]. According to surveys conducted by the American Association of Retired Persons [3] most of the 50+ population want to age in their homes and communities. In Israel about 97% of older people live in their homes in the community [4].

In recent decades, different types of specialized residential facilities for the older persons in the US have been developed, such as the “Naturally Occurring Retirement Community” (NORC), “Community Innovation for Aging in Place” (CIAIP), and “Program of All-Inclusive Care for the Elderly” (PACE) [5, 6]. Based on the World Health Organization [7, 8] age-friendly communities have been established in the US [9] and in Canada [10]. Many countries operate similar programs in the community, for example, Sweden [11], Finland, Germany, Japan [12], Spain [13], and UK [12, 14]. These programs are based on a multidisciplinary service team and provide medical and social services for the welfare of their members, such as monitoring health, an alarm button connected to a control center, meal service, shopping and accompaniment service, shuttle service, and legal advice.

A program that has been developed in Israel by Eshel (the association for planning and development of services for the aged in Israel) and the departments for social services is the “supportive community” [15]. The program is intended for the general older population at all levels of functioning who are living in both urban and rural settlements in any of the population sectors (Jewish, Arab, immigrant, etc.). The basket of services include the following: an emergency call-button and a 24-hour call center; medical services: a home visit by a doctor, 24/7 at a nominal fee and ambulance service even without a doctor's referral, also at a nominal fee; and social and cultural activities: lectures, exercise, cultural events, classes, parties, and excursions. The unique feature of the program is the “community parent” service. The community parent is the first contact for members for all their daily needs, from emergency situations to minor household repairs. For example, he offers help at home, such as smaller repairs like replacing a light bulb or moving furniture. For more complex repairs, he brings professionals and supervises their work. He helps in delivering prepared meals and medicines and in accompanying a member to the clinic/hospital. He also initiates telephone calls and home visits to the members, with the help of volunteers, and, if necessary, he contacts a family member—the caregiver. Currently, there are about 250 supportive communities in Israel with some 44,200 members (about 5.8% of those aged 65+). Every program is based on approximately 200 households. Membership fees are about $35 per month, and the needy older persons receive subsidies. Research results indicated that the program provided a solution for the target population; the majority of members expressed general satisfaction with the program and were satisfied with the services they received. The main reasons for joining the program were as follows: increased feeling of personal security, continued living at home, and relieving the burden of their care from family members [15, 16].

However, the family is still the main factor responsible for the care of older adults [17]. Eeven in large families, one family member, usually the spouse or one of the children, is a caregiver, that is, takes on the responsibility for the older person [17–19]. Primary caregivers are engaged in a variety of areas of assistance, such as personal care, financial assistance, housekeeping, and assistance with activities outside the home [17]. In many cases, there is also a pattern of partnership where most older people have a number of informal caregivers who can replace the primary caregiver when necessary, or a group of offspring divides between them the responsibility of providing practical help for the parent [20]. Szinovacz and Davey [21] found that daughters and children living closer to parents were more likely to remain primary caregivers.

Intensive caring for older parents may cause stress in primary caregivers, expressed in the process of losing their quality of life, causing damage to their physical and psychological health, and difficulties in their social and economic situation [22]. Burden can be caused because the caregiver sometimes has difficulty dealing with the demands of caregiving due to deterioration in the health of the aged parent, specifically care of older persons who suffer from cognitive impairment and behavioral problems burden [23]. Another source of the burden is family commitments. Adult children are called the “sandwich generation” because of the many obligations imposed on them by every generation in their family. Married primary caregivers experience a high sense of burden, especially those who shared a residence with the older parent [24]. Caregiving of older family members actually becomes another “career” among person's roles [25]. The unexpected career may cause difficulties in functioning at work, such as absenteeism, and, in extreme cases, even leaving the job [26]. In addition, caregiver obligations impinge upon the social domain by not enabling the caregiver to go on vacation or have recreation and leisure time [27]. Furthermore, care requirements that include a large number of hours of care and multiple tasks increase the burden [28]. Certain other socioeconomic characteristics of the caregiver have been found to affect the caregiver's burden:* gender*: most primary caregivers are women because of the traditional female role to take care of older parents, and they reported greater feeling of burden than men [29, 30],* age*: several studies found that advanced age of the primary caregiver was associated with a sense of burden [31] and other studies found younger primary caregivers reported more burden [32],* education*: whereas some studies reported that there is a connection between level of education and burden [29], others reported there is no such connection [33], and* religiosity*: some studies found that religious belief and religious rituals help to moderate the burden of the caregivers [34]. In contrast, coping strategies related to religiosity were not found to moderate the stress and depression of primary caregivers [35]. In addition, ethnic and cultural factors have been associated with caregivers' burden [28]. Although most of the studies dealt with the negative impact of caregiving on the quality of life of caregivers [36], studies over the past decade have reported the positive aspect in terms of finding meaning in the act of caregiving [37].

There are two main factors that help moderate the burden of primary caregivers. The first one is receiving specific help from another family member [38]. The second one is the assistance of formal systems [18, 39].

As stated, the supportive community program is a formal service and was developed to fill the needs of older adults who continue living in their homes. One of the reasons for joining the program was relieving family members of the burden of their care [15] but, to date, no research has examined whether the program helps reduce the caregivers' burden and whether it contributes to their quality of life. Thus, the aim of this study was to add the perspective of the offspring who are primary caregivers of older parents.

The research hypotheses were as follows.

**Hypothesis 1 .**

The caregiver burden of offspring who are the primary caregivers of their older parents who are members of a supportive community will be lower than that of the caregivers whose parents are nonmembers.

**Hypothesis 2 .**

The quality of life of offspring who are the primary caregivers of their older parents who are members of a supportive community will be higher than that of the caregivers whose parents are nonmembers.

**Hypothesis 3 .**

The extent and frequency of providing assistance will be lower among the offspring caregivers whose parents are members of a supportive community.

**Hypothesis 4 .**

In the entire sample an inverse correlation will be found between caregiving burden and quality of life so that the lower the caregiving burden, the higher the quality of life.

## 2. Method 

### 2.1. The Sample and Procedure: Data Collection

The research sample was a convenience sample of 119 participants. Of these, the research group included 55 offspring of parents who were members of a supportive community, and the comparison group included 64 offspring whose parents were nonmembers. All of the older parents were at a normal level of cognitive functioning. Data collection was conducted after receiving approval by the ethics committee at the School of Social Work in the university. Participants were given an explanation by means of a phone call about the purpose of the research and were assured that their responses were anonymous and would be used for research purposes only. The criterion for inclusion of participants for the study was offspring of older parents who defined themselves as primary caregivers of their older parents. Participants of the research group were identified through social workers of welfare departments and through community parents. They identified which of the offspring were the primary caregivers as whom they contacted when the involvement of a family member for the older parent was needed. Participants in the comparison group were identified through friends and networking applications of the research assistants. In the study group we succeeded in contacting 60 offspring who met the criteria to participate in the study but only 55 offspring agreed to participate in the study and fully filled out the questionnaire. In the comparison group we succeeded in contacting 70 offspring who met the criteria to participate in the study but only 64 offspring agreed to participate in the study and fully filled out the questionnaire. Those who were not included in the study also met the criteria but were not interested in participating in the study because of lack of time or lack of interest. Those who agreed to participate in the study filled out the questionnaires by themselves. All participants lived in central Israel.

### 2.2. Measures

#### 2.2.1. The Caregiving Burden Questionnaire

The short version of the Zarit burden interview [40] includes 12 items in its Hebrew version [41]. The questionnaire consists of two factors: (1) Personal stress factor, which includes nine items, for example, “Do you feel that because of the time you spend with your parent, you do not have enough time for yourself?” (2) Role stress factor, which includes three items, for example, “Do you feel you can take care of your parent in a better way?” Answers to the questions were on a five-point Likert scale: 1: “never” to 5: “almost always.” Each score was calculated based on the average of the total items. High score represents a greater feeling of caregiving burden. In our study the reliability for the entire scale was *α* = 0.79, for personal stress was *α* = 0.81, and for role stress was *α* = 0.55.

#### 2.2.2. Quality of Life Questionnaire

The questionnaire was developed by the World Health Organization Quality of Life Group [42] in its Hebrew version [43] and is a measurement tool for self-reported subjective perception of people about their quality of life. The questionnaire measures four domains: Physical health, for example, “How satisfied are you with your sleep?”; psychological health, for example, “How often do you have negative feelings such as blue mood, despair, anxiety, depression?”; social relationships, for example, “How satisfied are you with your personal relationships?” and the environment, for example, “How satisfied are you with your mode of transportation?” In the current study there were 26 questions, and participants had to answer questions related to their life during the previous two weeks. Answers to the questions were on a five-point Likert scale, where 1 represents the answers “very poor,” “not at all,” “not satisfied,” and “never” and 5 represents the answers “very good,” “largely very,” “very satisfied,” and “always”. Each area score is calculated based on the average of the total items. The higher the score, the higher the respondent's quality of life. The reliability of the questionnaire used in our study was *α* = 0.94. In addition, in this study we used ten expressions of emotions [43]: four positive items, like “excited” and “enthusiastic” and six negative items, like “worried” and “scared”. The participants had to answer to what extent the emotions were felt during the previous two weeks. Answers were on a five-point Likert scale when 1 indicates “not at all” and 5 “very great extent.” The reliability for positive items was *α* = 0.85 and the reliability for negative items was *α* = 0.55.

#### 2.2.3. Demographic Questionnaire

The questionnaire includes questions about gender, age, years of education, marital status, religiosity, employment, income status, and number of children. Questions about functional status of the older parents was rated by their offspring in accordance with three levels of Activity Daily Living (ADL) and Instrumental Activity Daily Living (LADL) as follows: independent—do not need help; frail—need partial help; and dependent—need full help. The offspring also answered the question about receiving homecare services under the Long Term Insurance Law (1: “yes” and 2: “no”), in addition, the offspring answered the questions concerning the caregiving of the parent, such as number of years of caregiving (1: “up to one year,” 2: “2-3 years,” and 3: “above 3 years”), number of days of caregiving per week (1: “every day,” 2: “several days a week,” and 3: “once a week”), and sharing in caregiving (1: “alone” and 2: “with family member”), and questions about the extent and frequency of caregiving assistance, such as housekeeping, transportation, shopping, financial management, and personal care and recreation and leisure (1: “does not provide assistance at all,” 2: “provides nonpermanent assistance,” and 3: “provides permanent assistance”).

### 2.3. Data Analysis

Differences in background characteristics were analyzed using chi-square and *t*-tests. Next, we analyzed the research hypotheses. *t*-tests for independent samples were calculated to examine differences between the two groups with regard to the following: (1) The total caregiver burden of offspring (12 items) and with regard to personal stress factor (9 items) and with regard to role stress factor (3 items). (2) The total quality of life (26 questions) of offspring and with regard to the four domains, physical health, psychological health, social relationships, and the environment, and to positive and negative emotions. In addition, Multivariate Analysis with Covariates test was conducted while controlling for age, gender, years of education, and functional status. *t*-test for independent samples was calculated to examine differences between the two groups with regard to the extent of help and frequency of actual assistance giving by offspring caregivers to their parents. Then, Spearman correlation was conducted to examine the relationship between the caregiver burden and quality of life of the offspring in the entire sample. Finally, linear regression models were constructed to examine the variables that predict the total caregiver burden variable and its factors and total quality of life and its domains.

## 3. Results

### 3.1. Sociodemographic Characteristics of the Sample

Table 1 presents the sociodemographic characteristics of the participants. Marital status groups were combined into two groups, married plus living with a partner and singles plus divorced and widowed, due to the small number of participants.

The data in Table 1 show a significant correlation between the functioning status of the parent with membership in supportive community. Of the 55 members of the supportive community 17 (30.9%) parents were functioning independently, 31 (56.4%) were frail parents, and 7 (12.7%) were dependent parents. Of the 64 who were not members, 30 (46.9%) parents were functioning independently, 19 (29.7%) were frail parents, and 15 (23.4%) were dependent parents. It should be noted that there is discrepancy between the percentage of those who are defined as frail and dependent and those who actually receive long term care services. The gap stems from the fact that the definition of the functional status of the older parents in our study was rated subjectively by their offspring while the benefits of long term insurance are based on tests. The National Insurance Institute sends professionals—a nurse or a physiotherapist—to examine the older person at home (an older person who is 90 years old or over may choose to undergo the functioning capacity examination by a gerontologist). Long term care services are provided only for those who are dependent on another for carrying out daily activities and not for those who only need assistance in managing a household. In addition, the entitlement depends on the income of an older person.

### 3.2. Caregiving Burden of Offspring, the Primary Caregivers

*t*-tests for independent samples were calculated to examine differences between the two groups.

Table 2 shows no differences between the groups in total caregiving burden. Examining the two factors of caregiving burden separately, a significant difference in role stress was found. Role stress burden among offspring of members of a supportive community was lower (M = 2.50, *N* = 55) than among offspring of nonmembers (M = 2.82, *N* = 64), *t* = −2.40, *p* < .05. In addition, a Multivariate Analysis with Covariates test was conducted while controlling for age, gender, years of education, and functional status. According to this analysis a significant difference was also found only with regard to the role stress burden (*F*(1,117) = 4.243, *p* < .05). Hypothesis 1 was partially confirmed.

### 3.3. Quality of Life among Offspring, the Primary Caregivers

*t*-tests for independent samples were calculated to examine differences between the two groups.

Table 3 shows that significant difference was found in the psychological domain of quality of life. Psychological health of offspring of members of a supportive community was higher (M = 4.00, *N* = 55) compared to offspring of nonmembers (M = 3.78, *N* = 64), *t* = 2.48, *p* < .05. With regard to the emotions variable, a significant difference was found in the negative emotions. Negative emotions of offspring of members of a supportive community were lower (M = 2.07, *N* = 55) compared to offspring of nonmembers (M = 2.45, *N* = 64), *t* = −2.67, *p* < .01. In addition, a Multivariate Analysis with Covariates test was conducted while controlling for age, gender, years of education, and functional status. According to this analysis a significant difference was also found only with regard to the psychological domain (*F*(1,117) = 6.742, *p* < .05). With regard to the emotions variable, a significant difference was also found only in the negative emotions, although the significance was lower (*F*(1,117) = 5.459, *p* < .05). Hypothesis 2 was confirmed regarding the psychological domain.

### 3.4. The Extent and Frequency of Caregiving

To examine whether there are differences between the groups in the extent and frequency of providing actual assistance to parents, *t*-tests for independent samples were calculated.

Table 4 indicates that, contrary to the hypothesis, offspring whose parents were supportive community members gave them more general assistance and helped them more in financial management and leisure activities and supported them more emotionally than offspring whose parents were nonmembers.

### 3.5. The Correlation between Burden and Quality of Life

According to the hypothesis, a negative significant correlation was found between the burden and total quality of life (*r* = −.42, *p* < .001, *N* = 119). The lower the burden was, the higher the offspring caregivers' quality of life was. In addition, a positive significant correlation has been found between the expression of negative emotions and burden (*r* = 0.37, *p* < .001, *N* = 119). The higher the burden was, the higher the expression of negative emotions was.

### 3.6. The Predictor Variables of Burden

In order to examine the predictor variables of the burden and each of its factors, Stepwise Regression analysis models were conducted. The regression model introduced sociodemographic characteristics of the offspring, as well as variables related to number of years of caregiving, number of days of care per week, sharing in caregiving, functional status of the parent, receiving homecare services from Long Term Insurance Law, and membership in a supportive community.

Table 5 indicates that the income status of the offspring entered in the first step explained 5.2% of the variance in total burden, and number of days of care per week entered in the second step added 3.6% to the explained variance. The total percentage of explained variance is 8.8%. That is, lower income and more days of care per week contribute to higher total burden of offspring.

The number of days of care per week entered in the first step explained 6.3% of the variance in personal stress factor of the offspring, and income status entered in the second step added 5.8% to the explained variance; religiosity entered in the third step added 3.4% to the explained variance, and receiving homecare services from Long Term Insurance Law entered in the fourth step added 2.9% to the explained variance. The total percentage of explained variance is 18.4%. That is, having lower income, being not religious, and not receiving homecare services for the parent contribute to the higher personal stress of offspring.

In addition, membership in a supportive community was the only variable which was entered to explain role stress, and the percentage of explained variance was 4.7%.

### 3.7. The Predictor Variables of Quality of Life

In order to examine the predictor variables of the quality of life and each of its domains, Stepwise Regression analyses models were conducted. The regression model introduced sociodemographic characteristics of the offspring, as well as variables related to burden, number of years of caregiving, number of days of care per week, sharing in caregiving, functional status of the parent, receiving homecare services from Long Term Insurance Law, and membership in supportive community.

Table 6 indicates that burden entered in the first step explained 16.2% of the variance in total quality of life, income status entered in the second step added 7.4% to the explained variance, and receiving homecare services from Long Term Insurance Law entered in the third step added 2.7% to the explained variance. The total percentage of explained variance is 26.3%. That is, low burden, having high income, and the parent receiving homecare services contribute to the total quality of life of offspring. Burden entered also in the first step explained 18.2% of the variance in physical quality of life, income status entered in the second step added 7.6% to the explained variance, and number of days of care per week entered in the third step added 2.8% to the explained variance. The total percentage of explained variance is 28.6%. That is, low burden, having high income, and less days of care per week contribute to the physical quality of life of offspring. Burden entered also in the first step explained 14.1% of the variance in psychological quality of life; age of caregiver entered in the second step added 5.3% to the explained variance. The total percentage of explained variance is 19.4%. That is, low burden and being younger contribute to the psychological quality of life of offspring. Burden entered also in the first step explained 11.4% of the variance in social relationships; gender of caregiver entered in the second step added 3.9% to the explained variance. The total percentage of explained variance is 15.3%. That is, low burden and being a women contribute to the social relationships of offspring. The income status of the offspring entered in the first step explained 13.3% of the variance in environment quality of life; sharing in caregiving added 3.6% to the explained variance. The total percentage of explained variance is 16.9%. That is, higher income status and sharing in caregiving contribute to the environment quality of life of offspring.

## 4. Discussion

This study examined the contribution of membership in a supportive community program to reducing the feeling of burden and increasing the quality life of offspring who are primary caregivers. The results of the study provided partial support for the hypotheses.

### 4.1. Feeling of Burden by Offspring Caregivers

The research hypothesis about the lower burden felt by offspring whose parents were members of a supportive community program has been confirmed regarding role stress, which consists of elements relating to the knowledge of how to take care of the older parent. Membership in the program allows offspring access to advice on professional care from a social worker, physician, or nurse whom they can consult regarding care requirements. Often this consultation takes place through the community parent who is usually the central figure in each program, and he is in close contact with the parent and aware of his condition [16]. Such consultation may contribute to reducing burden since these professionals are resources that can help bring relief to the caregiver [44]. Explaining why no significant differences were found between the groups with regard to feeling of total burden can focus on other variables that affect burden. The primary predictor of total burden and personal stress factor was income status, which also affects the total quality of life. This finding is supported by the meta-analysis conducted by Pinquart and Sörensen [22], whereby having higher income was related to better physical health of caregivers. It seems, therefore, that the feeling of burden is caused by pressures on the caregiver. He must fulfill different roles in stressful situations resulting from conflicts that exist between caregiving duties and the demands of employment [26]. Sharing in caregiving and fewer days of caregiving per week have been also been found to reduce feelings of burden. These findings are supported by previous studies that emphasized the importance of partnership of family members with the caregiver [38]. In addition, some formal community services such as receiving homecare help the caregivers to cope with their roles. In Israel a Long Term Insurance Law was enacted in 1986 to enable older people to age in place and was also intended to help the family members in caregiving. According to this law, frail older persons receive personal care and home help by homecare workers and can also visit in day care centers [1, 18]. In accordance with previous studies [18, 44], our study shows that this variable was a predictor of personal stress; that is, accepting such assistance through a homecare worker or visits in day care centers can reduce caregiver's burden. An additional formal resource is a supportive community program which is a predictor, although in low percentage, of the pressure added by role stress. This finding is similar to the difference found between the groups, and it can be concluded that membership in a supportive community helps reduce the offspring caregivers' burden.

### 4.2. Quality of Life

As expected, an inverse correlation has been found between burden and quality of life, so the lower the burden, the higher the quality of life among offspring caregivers. An examination of the differences in quality of life between the two groups found higher psychological quality of life among offspring whose parents were members, and they also expressed less negative emotions. The importance of the caregiver's psychological health was revealed in Pinquart and Sörensen's meta-analysis study [45] on differences between caregivers and noncaregivers. Their findings indicated that the largest difference between these two groups was with regard to depression symptoms while the smallest difference was in the dimension of physical health. That is to say, the burden has a far greater influence on the psychological health than on the physical health of primary caregivers. Our findings were also supported by the previous study which was conducted by Iecovich [18] in Israel. She found that the psychological quality of life of caregivers (e.g., spouses, offspring, daughters-in-law, and sons-in-law) was higher among those who care for an older family member who attended a day care center. Adult day care services center offers additional activities for older adults and interventions that help the caregivers in coping with their role and contribute to their overall well-being [39]. Similarly, a supportive community program which includes activities organized in a social club allows the older parent to participate in activities outside the home, as well as receiving in-home services [15]. These services provide help and partnership to caregivers and contribute especially in raising the psychological quality of life. However, burden linked with a negative perception of the quality of life of offspring who are primary caregivers [36]. Our research showed the same results; the regression analysis showed that burden affects quality of life.

### 4.3. Help Provided by the Offspring Caregivers to Their Parents

Contrary to the hypothesis, the extent and frequency of general assistance and some other areas of assistance were higher among offspring whose parents were members of a supportive community. A possible explanation for these results is related to parents' functioning, as most frail parents (31 of 50) were members while a minority (19) were nonmembers. Hence, offspring's assistance reflected the condition of the parents who need help and most of them were members. But, in other areas of assistance such as household chores, transportation, and personal care, no differences were found between the groups. Therefore, we can conclude that the filial obligation of offspring is important in modeling their behavior towards parents even if some assistance is received from formal services. Family members are still considered a primary resource for care of the older persons, and offspring have the opportunity to repay the love they received from their parents and provide them with the assistance they need [46, 47].

However, the main focus of our study results is that membership of older parents in supportive community programs contributed to the quality of life of their offspring who are primary caregivers, mainly psychologically. Membership of older parents in supportive community also reduces the feelings of burden particularly related to role stress burden. Therefore it can be concluded that the supportive community project provides a meaningful contribution to the offspring caregivers.

This study has two main limitations. The first limitation refers to the sampling process. We used a convenience and not a large sample which therefore may not be generalizable to all offspring primary caregivers whose older parents are members of a supportive community program. It is important to further examine the point of view of the offspring primary caregivers of older parents who receive this service. The second limitation is that the study examined the questions at one point of time. It would be worthwhile to expand the research study which will review the contribution of the program to reducing burden and increasing the quality of life of the caregivers offspring at several points in time: before membership in the program, after half a year, and after a year. Also, it would be worthwhile to include more questions eliciting the offspring's opinions about the various services provided by the program and their satisfaction with the degree of connection maintained with them as the primary caregivers.

## 5. Conclusions

The various services that were developed in the community and available for the older persons do not replace the family members' caregiving but help the caregivers. The findings of the present study indicate some contribution of the supportive community program to the well-being of the offspring primary caregivers. That is, the role stress factor of caregiving burden was lower, and the psychological health domain of quality of life was higher among offspring whose parents were members of supportive communities. Therefore, the practical conclusion of this study is to further develop and market supportive community programs in various communities and to enable the older adults to participate in the program.

## Figures and Tables

**Table 1:** Study sample.

Variable	Characteristic	Offspring of supportive community members (*N* = 55)		Offspring of not supportive community members (*N* = 64)		*χ* ^2^	*t*
		*N* (%)	M	*N* (%)	M		
Age (average, SD)			51.2, 7.319		50.1, 11.079		.664

Gender	Men	19 (34.5)		16 (25.0)		1.298
	Women	36 (65.5)		48 (75.0)		

Marital status	Married	39 (70.9)		48 (75.0)		.252
	Unmarried	16 (29.1)		16 (25.0)		

Years of education			13.4, 3.035		14.7, 2.561		2.39^*∗*^

Level of religiosity	Secular	24 (43.6)		34 (53.1)		1.866
	Traditional	21 (38.2)		17 (26.6)		
	Religious	10 (18.2)		13 (20.3)		

Employment status	Employed	46 (83.6)		59 (92.2)		2.084
	Unemployed	9 (16.4)		5 (7.8)		

Income	Below average	17 (30.9)		24 (37.5)		4.844
	Average	25 (45.5)		17 (26.6)		
	Above average	13 (23.6)		23 (35.9)		

Functional status of the parent	Independent	17 (30.9)		30 (46.9)		8.754^*∗*^
	Frail	31 (56.4)		19 (29.7)		
	Dependent	7 (12.7)		15 (23.4)		

Number of days of caregiving per week	Every day	6 (10.9)		12 (18.7)		3.021
	Several days a week	24 (43.6)		19 (29.7)		
	Once a week	25 (45.5)		33 (51.6)		

Number of years of caregiving	Up to one year	14 (25.4)		22 (34.4)		2.163
	2-3 years	15 (27.3)		11 (17.2)		
	Above 3 years	26 (47.3)		31 (48.4)		

Sharing in caregiving	Alone	19 (34.5)		17 (26.6)		1.147
	With family member	36 (65.5)		47 (73.4)		

Receiving homecare services under Long Term Insurance Law	Yes	26 (47.3)		21 (32.8)		2.588
	No	29 (52.7)		43 (67.2)		

^*∗*^ *p* < .05.

**Table 2:** Differences in burden between offspring of supportive community members (*N* = 55) and nonmembers (*N* = 64).

Variable	Factor	Group (offspring of parents of supportive community)	M	SD	*t*
Total burden		Members Nonmembers	2.05 2.18	.479 .537	−1.46
Burden	Personal stress	Members Nonmembers	1.89 1.97	.427 .618	−.73
Burden	Role stress	Members Nonmembers	2.50 2.82	.693 .724	−2.40^*∗*^

^*∗*^ *p* < .05.

**Table 3:** Differences in quality of life between offspring of supportive community members (*N* = 55) and nonmembers (*N* = 64).

Variable	Factor	Group (offspring of parents of supportive community)	Mean	SD	*t*
Total quality of life		Members	3.84	.450	1.18
		Nonmembers	3.74	.461	
Quality of life	Physical health	Members	3.86	.557	.60
		Nonmembers	3.80	.536	
Quality of life	Psychological health	Members	4.00	.472	2.48^*∗*^
		Nonmembers	3.78	.487	
Quality of life	Social relationships	Members	3.71	.765	.48
		Nonmembers	3.65	.627	
Quality of life	Environment	Members	3.72	.478	−.07
		Nonmembers	3.73	.642	
Emotions	Positive emotions	Members	2.52	.691	−1.07
		Nonmembers	2.66	.704	
Emotions	Negative emotions	Members	2.07	.771	−2.67^*∗∗*^
		Nonmembers	2.45	.786	

^*∗*^ *p*< .05; ^*∗∗*^ *p*< .01.

**Table 4:** Differences in the extent and frequency of care between offspring of supportive community members (*N* = 55) and nonmembers (*N* = 64).

Variable	Group (offspring of parents of supportive community)	Mean	SD	*t*
General assistance	Members Nonmembers	3.17 2.72	.693 .730	3.41^*∗∗∗*^

Number of days per week	Members Nonmembers	2.34 2.32	.672 .777	.12

Household chores	Members Nonmembers	2.60 2.34	1.69 1.42	.89

Transportation and/or shopping	Members Nonmembers	3.43 3.12	1.06 1.37	1.18

Personal care	Members Nonmembers	1.76 1.56	.130 .973	.96

Financial management	Members Nonmembers	3.65 2.53	1.39 1.58	4.08^*∗∗∗*^

Financial assistance	Members Nonmembers	2.41 1.93	1.37 1.46	1.83

Emotional support	Members Nonmembers	4.92 4.34	.539 1.12	3.50^*∗∗∗*^

Leisure activities	Members Nonmembers	4.12 3.53	1.07 1.24	2.77^*∗∗∗*^

^*∗∗∗*^ *p* < .001.

**Table 5:** Stepwise Regression predicting burden.

Model	Variables entered	*B*	SE	*β*	*t*	*R* ^2^
Total burden						
Step 1	Income	−.145	.057	−.227	−2.53^*∗*^	.052
Step 2	Income	−.149	.057	−.234	−2.63^*∗*^	.088
	Number of days of caregiving per week	−.134	.063	−.189	−2.13^*∗*^	
Personal stress burden						
Step 1	Number of days of caregiving per week	−.199	.071	−.250	−2.80^*∗∗*^	.063
Step 2	Number of days of caregiving per week	−.205	.069	−.259	−2.97^*∗∗*^	.121
	Income	−.174	.062	−.242	−2.78^*∗∗*^	
Step 3	Number of days of caregiving per week	−.208	.068	−.262	−3.06^*∗∗*^	.155
	Income	−.183	.062	−.256	−2.98^*∗∗*^	
	Religiosity	−.137	.064	−.183	−2.13^*∗*^	
Step 4	Number of days of caregiving per week	−.185	.068	−.234	−2.72^*∗∗*^	.184
	Income	−.170	.061	−.237	−2.78^*∗∗*^	
	Religiosity	−.143	.063	−.192	−2.26^*∗*^	
	Receiving homecare services	−.205	.102	−.174	−2.02^*∗*^	
Role stress burden						
Step 1	Membership in supportive community	.314	.131	.217	2.40^*∗*^	.047

^*∗*^ *p* < .05; ^*∗∗*^ *p* < .01.

**Table 6:** Stepwise Regression predicting quality of life.

Model	Variables entered	*B*	SE	*β*	*t*	*R* ^2^
Total QoL						
Step 1	Total burden	−.358	.075	−.402	−4.75^*∗∗∗*^	.162
Step 2	Total burden	−.301	.074	−.339	−4.06^*∗∗∗*^	.236
	Income	.158	.047	.280	3.35^*∗∗∗*^	
Step 3	Total burden	−.331	.075	−.372	−4.44^*∗∗∗*^	.263
	Income	.164	.047	.290	3.52^*∗∗∗*^	
	Receiving homecare services	−.158	.076	−.170	−2.07^*∗*^	
Physical QoL						
Step 1	Total burden	−.453	.089	−.427	−5.10^*∗∗∗*^	.182
Step 2	Total burden	−.384	.087	−.363	−4.41^*∗∗∗*^	.258
	Income	.191	.055	.282	3.43^*∗∗∗*^	
Step 3	Total burden	−.348	.087	−.329	−3.98^*∗∗∗*^	.286
	Income	.200	.055	.296	3.64^*∗∗∗*^	
	Number of days of caregiving per week	.128	.060	.170	2.12^*∗*^	
Psychological QoL						
Step 1	Total burden	−.359	.082	−.376	−4.39^*∗∗∗*^	.141
Step 2	Total burden	−.357	.080	−.374	−4.48^*∗∗∗*^	.194
	Age of caregiver	.012	.004	.229	2.74^*∗∗*^	
Social relationships QoL						
Step 1	Total burden	−.454	.117	−.337	−3.87^*∗∗∗*^	.114
Step 2	Total burden	−.452	.115	−.336	−3.93^*∗∗∗*^	.153
	Gender of caregiver	.299	.129	.197	2.31^*∗*^	
Environment QoL						
Step 1	Income	.258	.061	.365	4.23^*∗∗∗*^	.133
Step 2	Income	.257	.060	.363	4.29^*∗∗∗*^	.169
	Sharing in caregiving	.156	.069	.191	2.26^*∗*^	

Note: QoL = Quality of Life, ^*∗*^ *p* < .05, ^*∗∗*^ *p* < .01, and ^*∗∗∗*^ *p* < .001.
